# Early Natural Stimulation through Environmental Enrichment Accelerates Neuronal Development in the Mouse Dentate Gyrus

**DOI:** 10.1371/journal.pone.0030803

**Published:** 2012-01-25

**Authors:** Na Liu, Shan He, Xiang Yu

**Affiliations:** 1 Institute of Neuroscience and State Key Laboratory of Neuroscience, Shanghai Institutes for Biological Sciences, Chinese Academy of Sciences, Shanghai, China; 2 Graduate School of the Chinese Academy of Sciences, Shanghai, China; Nathan Kline Institute and New York University School of Medicine, United States of America

## Abstract

The dentate gyrus is the primary afferent into the hippocampal formation, with important functions in learning and memory. Granule cells, the principle neuronal type in the dentate gyrus, are mostly formed postnatally, in a process that continues into adulthood. External stimuli, including environmental enrichment, voluntary exercise and learning, have been shown to significantly accelerate the generation and maturation of dentate granule cells in adult rodents. Whether, and to what extent, such environmental stimuli regulate the development and maturation of dentate granule cells during early postnatal development is largely unknown. Furthermore, whether natural stimuli affect the synaptic properties of granule cells had been investigated neither in newborn neurons of the adult nor during early development. To examine the effect of natural sensory stimulation on the dentate gyrus, we reared newborn mice in an enriched environment (EE). Using immunohistochemistry, we showed that dentate granule cells from EE-reared mice exhibited earlier morphological maturation, manifested as faster peaking of doublecortin expression and elevated expression of mature neuronal markers (including NeuN, calbindin and MAP2) at the end of the second postnatal week. Also at the end of the second postnatal week, we found increased density of dendritic spines across the entire dentate gyrus, together with elevated levels of postsynaptic scaffold (post-synaptic density 95) and receptor proteins (GluR2 and GABA_A_Rγ2) of excitatory and inhibitory synapses. Furthermore, dentate granule cells of P14 EE-reared mice had lower input resistances and increased glutamatergic and GABAergic synaptic inputs. Together, our results demonstrate that EE-rearing promotes morphological and electrophysiological maturation of dentate granule cells, underscoring the importance of natural environmental stimulation on development of the dentate gyrus.

## Introduction

The dentate gyrus is one of two main locations of continuous neurogenesis in the adult rodent brain [1], [2]. It is the primary afferent into the hippocampus, a medial cortical structure required for the formation of new memories and some forms of learning behavior [3], [4]. Classical anatomical studies revealed the general developmental plan of the dentate gyrus [5], [6], [7], [8]. In rodents, 80% of granule cells detected at 1 month of age are born postnatally, largely during the first week after birth [7], [9]. Granule cells continue to be born in the subgranular zone of the dentate gyrus throughout the animal's lifetime.

Many genes have been identified to play critical roles during distinct stages of dentate gyrus formation and development [10], [11]. Of mutants that survived into adulthood, mice lacking the transcription factor NeuroD, with relatively specific defects in the differentiation of dentate granule cells [12], [13], displayed spontaneous limbic seizures [13]. In another example, mice mutant for Frizzled 9, a gene in the Williams syndrome deletion interval, with increased apoptosis of dentate granule cells during development, had diminished seizure threshold and severe deficits in tests of visuospatial learning and memory [14]. These examples pinpoint the importance of dentate gyrus development for normal brain function and plasticity.

External stimuli, including environmental enrichment, voluntary exercise, and hippocampal-dependent learning have been demonstrated to significantly accelerate the genesis and maturation of dentate granule cells in adult rodents [15], [16], [17], [18]. Whether these stimuli also affect neuronal maturation during early development remains largely unknown. Furthermore, since most studies of activity-dependent neurogenesis in the adult have focused on the rate of new neuron formation and on the morphological maturation of neurons, a detailed study that encompasses both functional and morphological aspects of activity-induced neuronal maturation in the dentate gyrus would be interesting and important to studies of both hippocampal development and adult neurogenesis. This is especially interesting because the course of neuronal maturation in the adult hippocampus has been shown to recapitulate that of early development [19], albeit with a slower time course [20], [21], [22]. Thus, a better understanding of activity-dependent regulation of neuronal maturation during early development would also shed light on such events in the mature animal.

To examine the effect of natural sensory stimulation on the development of dentate granule cells in early postnatal animals, we reared newborn mice in an enriched environment (EE), a paradigm that promoted neuronal activity through a “combination of complex inanimate and social stimulation” [23], [24]. Using immunohistochemical, biochemical and electrophysiological assays, we showed that this paradigm significantly accelerated morphological and functional maturation of dentate granule cells during the first two postnatal weeks, slowly plateauing by the end of the third postnatal week. These results demonstrate the importance of natural environmental stimulation in promoting multiple aspects of dentate granule neuron development and maturation.

## Results

### Accelerated morphological maturation of dentate granule cells by EE-rearing

**Figure 1:**
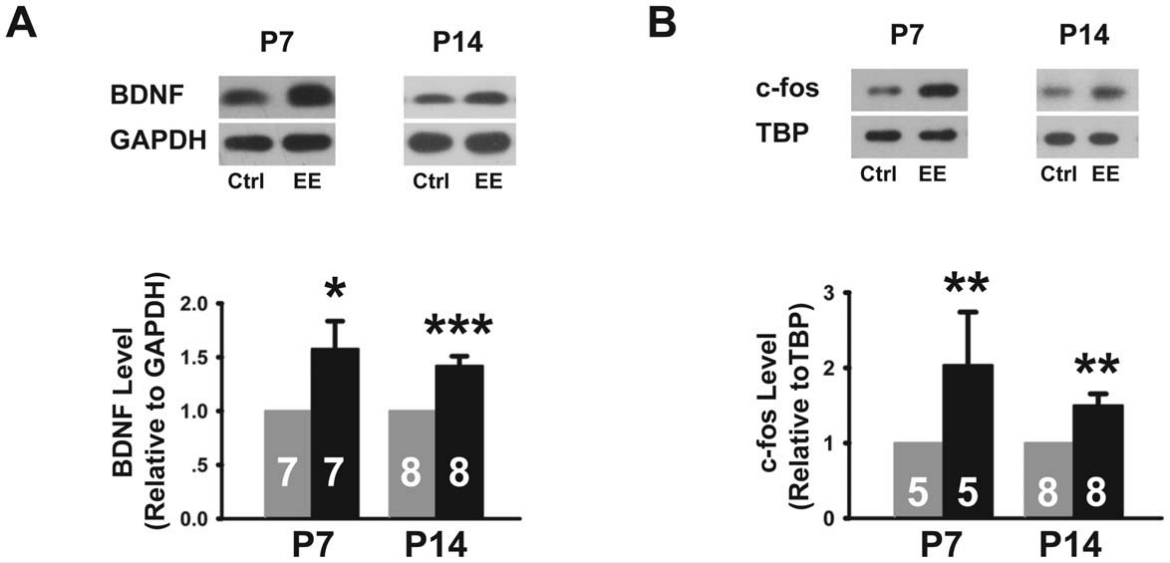
Dentate granule cells in P7 and P14 EE-reared mice responded to increases in neuronal activity. (A) Immunoblot and quantitation showing elevated level of BDNF in dentate gyrus preparations of P7 and P14 EE-reared mice (P7: 1.58±0.26, *P*<0.05; P14: 1.42±0.09, *P*<0.001). (B) Immunoblot and quantitation showing increased level of c-fos in nuclear fractions from the dentate gyrus of P7 and P14 EE-reared mice (P7: 2.03±0.71, *P*<0.01; P14: 1.50±0.15, *P*<0.05). Error bars represent s.e.m., and “*n*”, as indicated inside bar graphs, represents the number of age-matched litter pairs; *P<0.05, **P<0.01, ***P<0.001.

To determine whether our environmental enrichment paradigm significantly elevated neuronal activity in the dentate gyrus of newborn mice, we assayed the protein level of two well known activity-induced genes, namely brain-derived neurotrophic factor BDNF [25], [26], [27] and the immediate early gene c-fos [28]. The level of both proteins was found to be significantly elevated in extracts from the dentate gyrus of P7 and P14 EE-reared mice, as compared to those reared in standard control cages (Figure 1), demonstrating the effectiveness of our paradigm in elevating neuronal activity *in vivo*.

**Figure 2:**
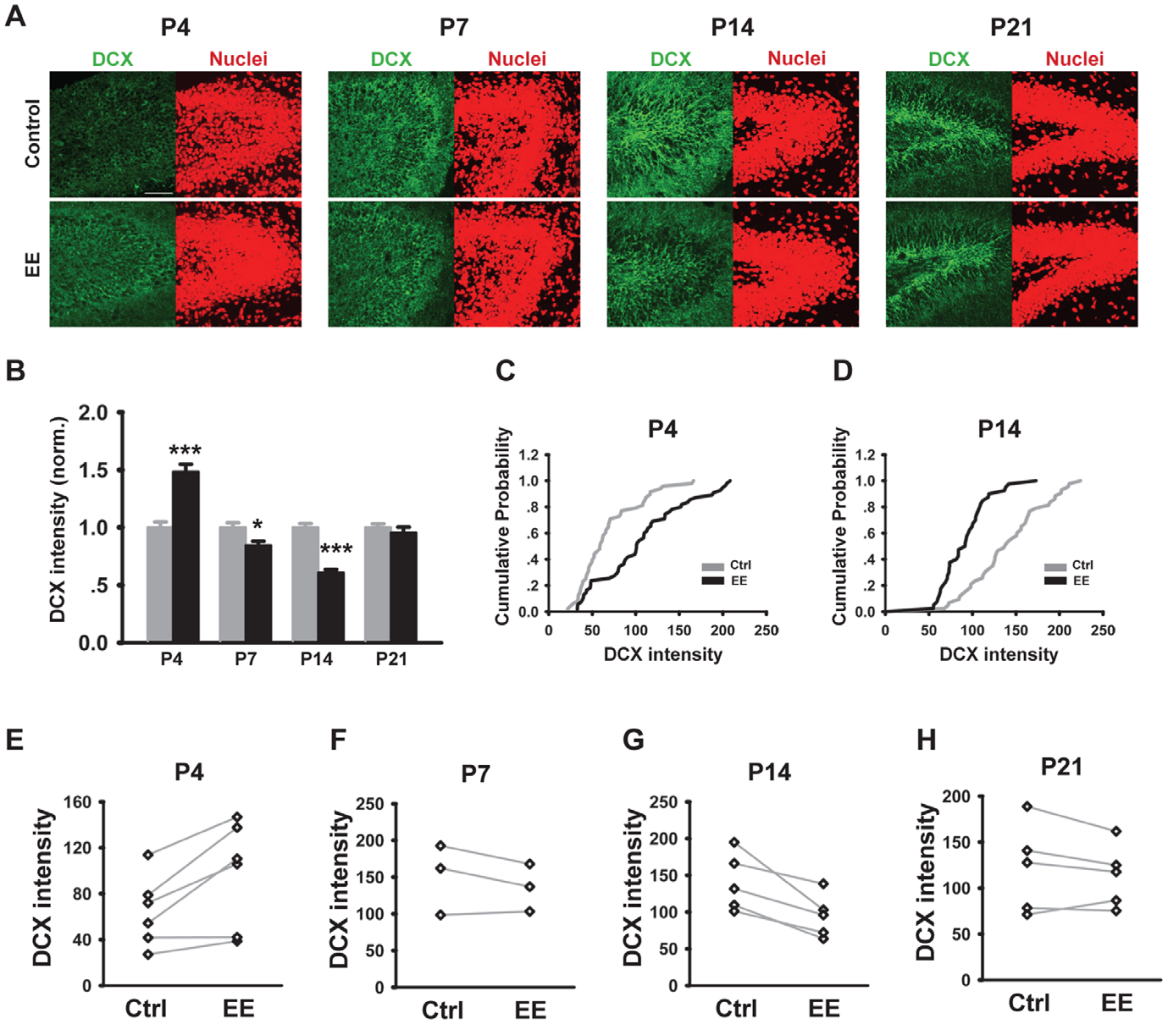
EE-rearing accelerated the time course of DCX expression in granule cells of the dentate gyrus. (A) Example images of coronal sections through the dentate gyrus immunostained with DCX antibody (green channel) and TO-PRO-3 (nuclei, red channel). Mice are from different postnatal ages and reared in control or EE conditions as indicated. Scale bar is 50 µm. (B) Quantitation of DCX fluorescence intensity normalized to TO-PRO-3. EE-rearing significant increased DCX expression at P4 (1.49±0.06, N = 6 mice each, *n* = 48 brain slices for Ctrl and 55 for EE, *P*<0.001) and reduced it at P7 (0.91±0.03, N = 3 mice each, n = 24 each, *P*≤0.05) and at P14 (0.65±0.02, N = 5 mice each, *n* = 43 for Ctrl and 40 for EE, *P*<0.001). Gray bars represent the control condition, black bars represent EE-rearing; error bars represent s.e.m. (C–D) Cumulative probability plots showing significant increase in DCX fluorescence intensity in P4 EE-reared mice (C, *P*<0.001) and significant reduction at P14 (D, *P*<0.001). (E–H) Graphs showing changes in DCX intensity between pairs of co-processed Ctrl and EE mice, age as indicated. *P≤0.05, ***P<0.001.

We then proceeded to examine the effect of early postnatal EE-rearing on the morphological maturation of dentate granule cells, which follow a well-characterized and stereotypic time course [1], [2], [11], [29]. Doublecortin (DCX) is a microtubule-associated protein expressed by neuronal precursor cells and immature neurons [30], [31], [32], often used as a marker of young neurons [33]. At P4, the earliest time point at which we were able to immunostain for DCX in control animals, we observed significantly elevated level of DCX in EE-reared mice as compared to controls (Figure 2A–C, E), consistent with the presence of more mature neurons. Interestingly, the level of DCX in EE-reared mice, as compared to controls, was reduced at P7, an effect that persisted until P14 and leveled by P21 (Figure 2). This result was somewhat surprising at first glance, as from our previous work [34], we expected more neurons or more mature neurons in EE-reared mice. On further thought, taking into account the transient nature of DCX expression in newly generated neurons [33], we surmised that accelerated neuronal maturation could actually result in earlier disappearance of DCX expression.

**Figure 3:**
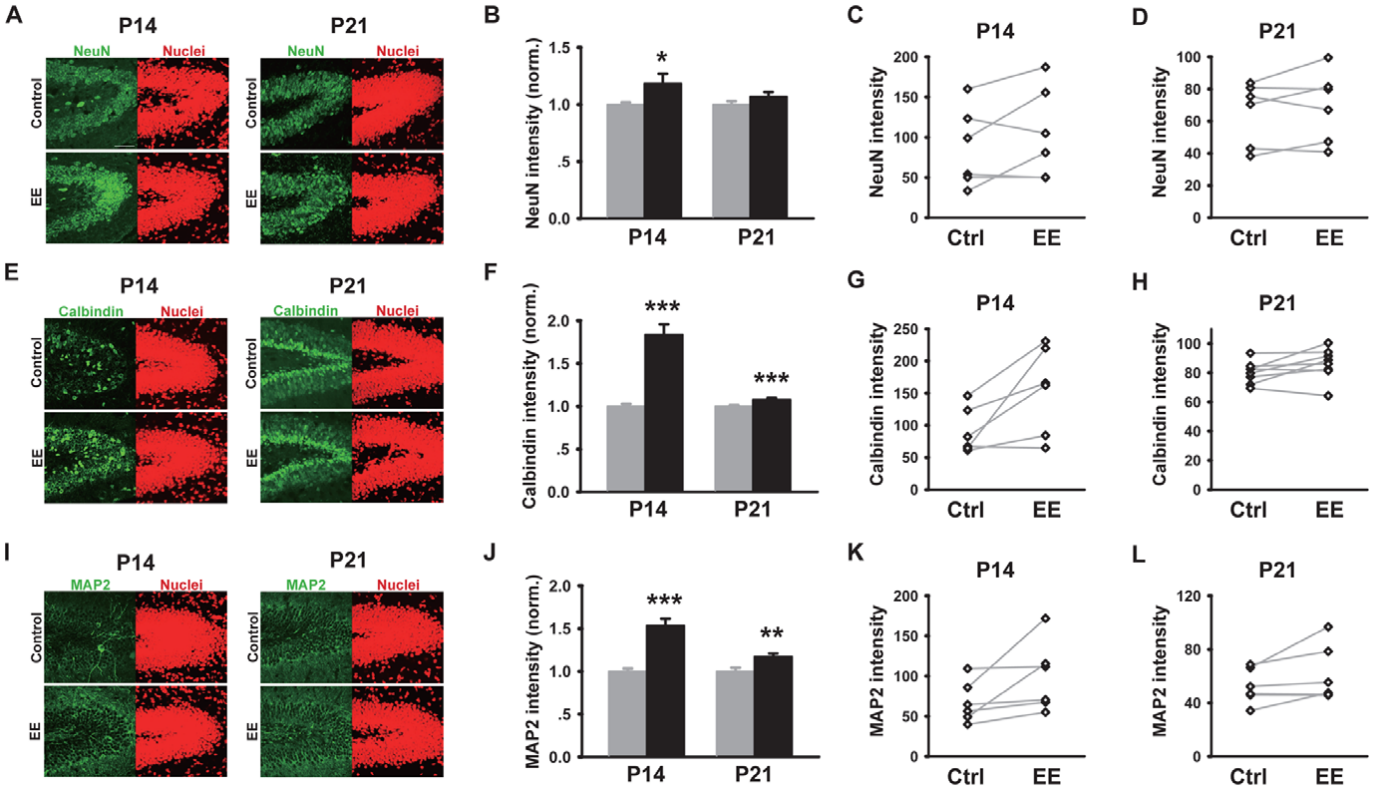
Increased expression of mature neuronal markers in dentate granule cells of EE-reared mice. (A, E, I) Example images of dentate gyrus sections immunostained with NeuN (A), calbindin (E) or MAP2 (I) and counterstained with TOPRO3, age and rearing condition as indicated. Scale bar is 50 µm. (B) Quantitation showing increased NeuN expression in EE-reared mice at P14 (1.18±0.09, N = 6 mice each, *n* = 51 for Ctrl and 40 for EE, *P*<0.05). (C–D) Graph showing changes in NeuN expression between pairs of co-processed Ctrl and EE mice, age as indicated. (F) Calbindin expression was significantly increased in EE-reared mice at P14 (1.84±0.12, N = 6 mice each, *n* = 51 for Ctrl and 54 for EE, *P*<0.001), and continued to be so at P21 (1.08±0.02, N = 8 mice each, *n* = 73 for Ctrl and 78 for EE, *P*<0.001). (G–H) Graph showing changes in Calbindin expression between pairs of co-processed Ctrl and EE mice, age as indicated. (J) EE-rearing significantly increased MAP2 immunoreactivity in P14 mice (1.54±0.08, N = 6 mice each, *n* = 56 for Ctrl and 53 for EE, *P*<0.001), an effect that continued into P21 (1.17±0.04, N = 6 mice each, *n* = 58 for Ctrl and 46 for EE, *P*<0.01). (K–L) Graph showing changes in MAP2 expression between pairs of co-processed Ctrl and EE mice, age as indicated. *P<0.05, **P<0.01, ***P<0.001.

To test our hypothesis and further examine the effect of early EE-rearing on neuronal maturation, we assayed, using immunohistochemistry, the expression level of a number of mature neuronal markers, including NeuN, calbindin and microtubule-associated protein MAP2. NeuN is a neuron-specific nuclear protein, whose expression initially overlaps with DCX and persists through the life of mature neurons, long after DCX becomes undetectable [33], [35], [36]. At P14, we observed a small but significant increase in the level of NeuN immunoreactivity in EE-reared mice as compared to controls (Figure 3A–C), an effect that leveled by P21 (Figure 3A,B, D). Calbindin is a calcium-binding protein expressed in mature granule cells of the dentate gyrus [37], [38]. Our results demonstrated a significant increase in the level of calbindin expression in P14 EE-reared mice, as compared to controls (Figure 3E–G). Similar results were obtained for the expression of MAP2 [39], a neuronal-specific and predominantly dendritically localized cytoskeleton protein (Figure 3I–K). The effect of EE-rearing on promoting neuronal maturation are not limited to the dentate gyrus, as we also observed significant increase in MAP2 expression in CA1 region of the hippocampus in EE-reared mice at P14 (Figure S1A, B). By P21, the effect of EE-rearing in the dentate gyrus on calbindin and MAP2 expression were smaller in magnitude as compared with P14 (Figure 3E–F, H–J, L). In summary, the results of NeuN, calbindin and MAP2 immunohistochemistry (Figure 3), together with that of DCX (Figure 2), suggest accelerated maturation of dentate granule cells in EE-reared mice.

### Accelerated functional maturation of dentate granule cells by EE-rearing

**Figure 4:**
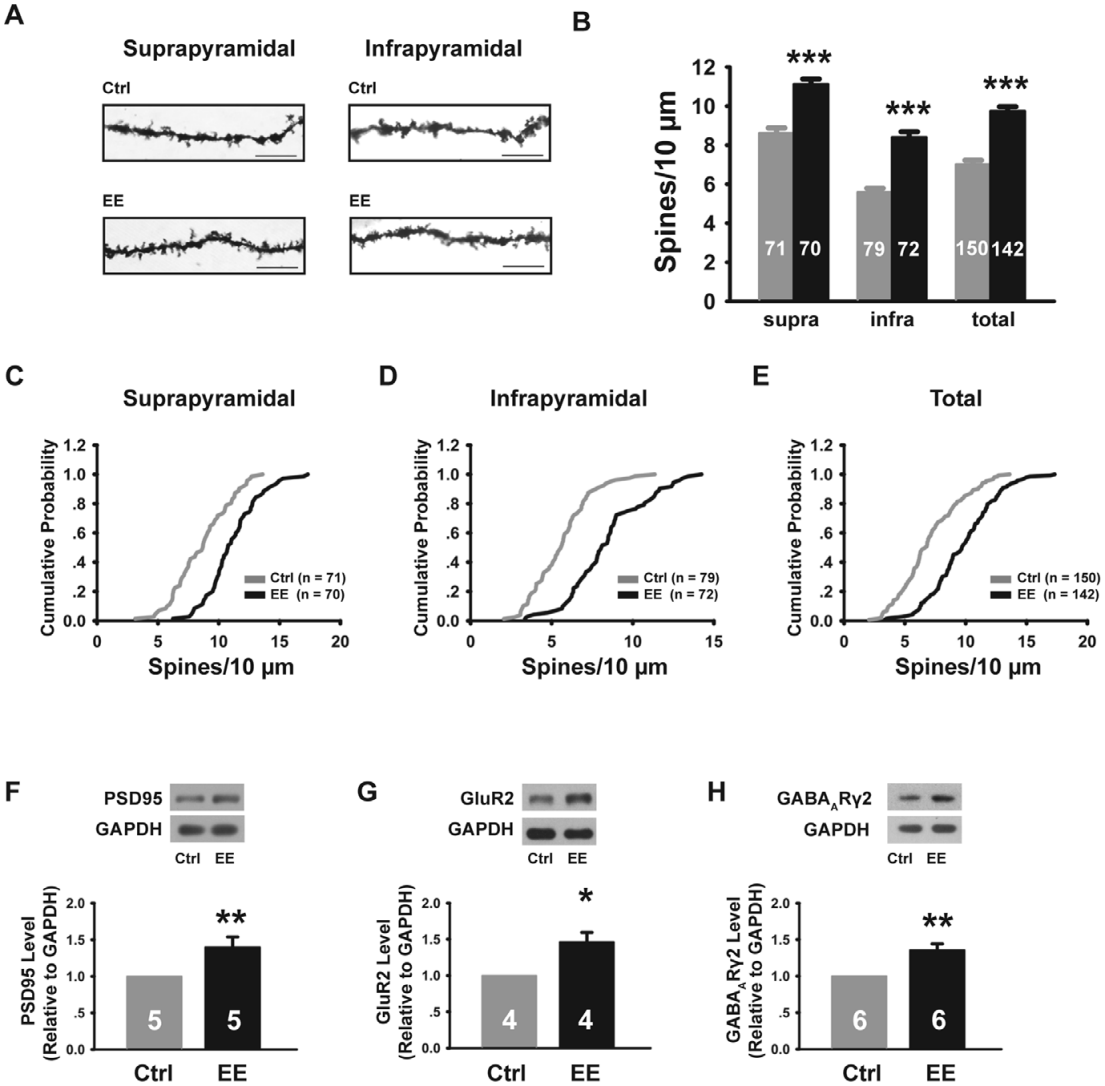
EE-rearing increased spine density and upregulated the level of postsynaptic proteins. (A) Example images of Golgi-stained granule cell dendrites, scale bar is 10 µm. (B) Dentate granule cells of EE-reared mice had significantly higher spine density as compared to control mice in both the suprapyramidal blade (Ctrl: 8.60±0.28; EE: 11.12±0.26, N = 6 mice each, *P*<0.001), the infrapyramidal blade (Ctrl: 5.58±0.20, EE: 8.40±0.28, N = 6 mice each, *P*<0.001), as well as when all cells were combined (Ctrl: 7.01±0.21, EE: 9.74±0.22, *P*<0.001), resulting in right shifts of the cumulative distribution plots (C–E, *P*<0.001 for all). (F–H) Immunoblots and quantitation of excitatory and inhibitory synaptic components from membrane fractions of the dentate gyrus. EE-rearing significantly increased the level of PSD95 (F, 1.40±0.14, *P*<0.01), GluR2 (G, 1.46±0.13, *P*<0.05) and GABA_A_Rγ2 (H, 1.36±0.09, *P*<0.01). Error bars represent s.e.m., and “*n*”, as indicated inside bar graphs, represents the number of neurons analyzed (B–E), or the number of age-matched litter pairs (F–H), *P<0.05, **P<0.01, ***P<0.001.

To determine if the above observed changes in the morphological maturation of dentate granule cells altered the function of these neurons, we first examined the effect of EE-rearing on the density of dendritic spines, the morphological bases of excitatory synapses. Since P14 was the developmental stage at which we observed the most significant morphological effects of EE-rearing, we focused on this developmental stage for the rest of the study. Measuring spine density in secondary dendrites, we found significant increases in granule cell spine density of EE-reared mice in both the suprapyramidal and infrapyramidal blades of the dentate gyrus at P14 (Figure 4A–E), consistently with the presence of more excitatory synapses. Similar increases in spine density were also observed in CA1 pyramidal neurons (Figure S1 C,D).

To assay whether the increase in spine density is accompanied by changes in the level of postsynaptic receptor and scaffold components, we performed Western blots using membrane fractions from the dentate gyrus. We found significant increases in the protein level of the excitatory scaffold protein post-synaptic density 95 (PSD95), AMPA receptor subunit GluR2 and GABA_A_ receptor (GABA_A_R) subunit γ2 (Figure 4F–H) in EE-reared mice at P14.

**Figure 5:**
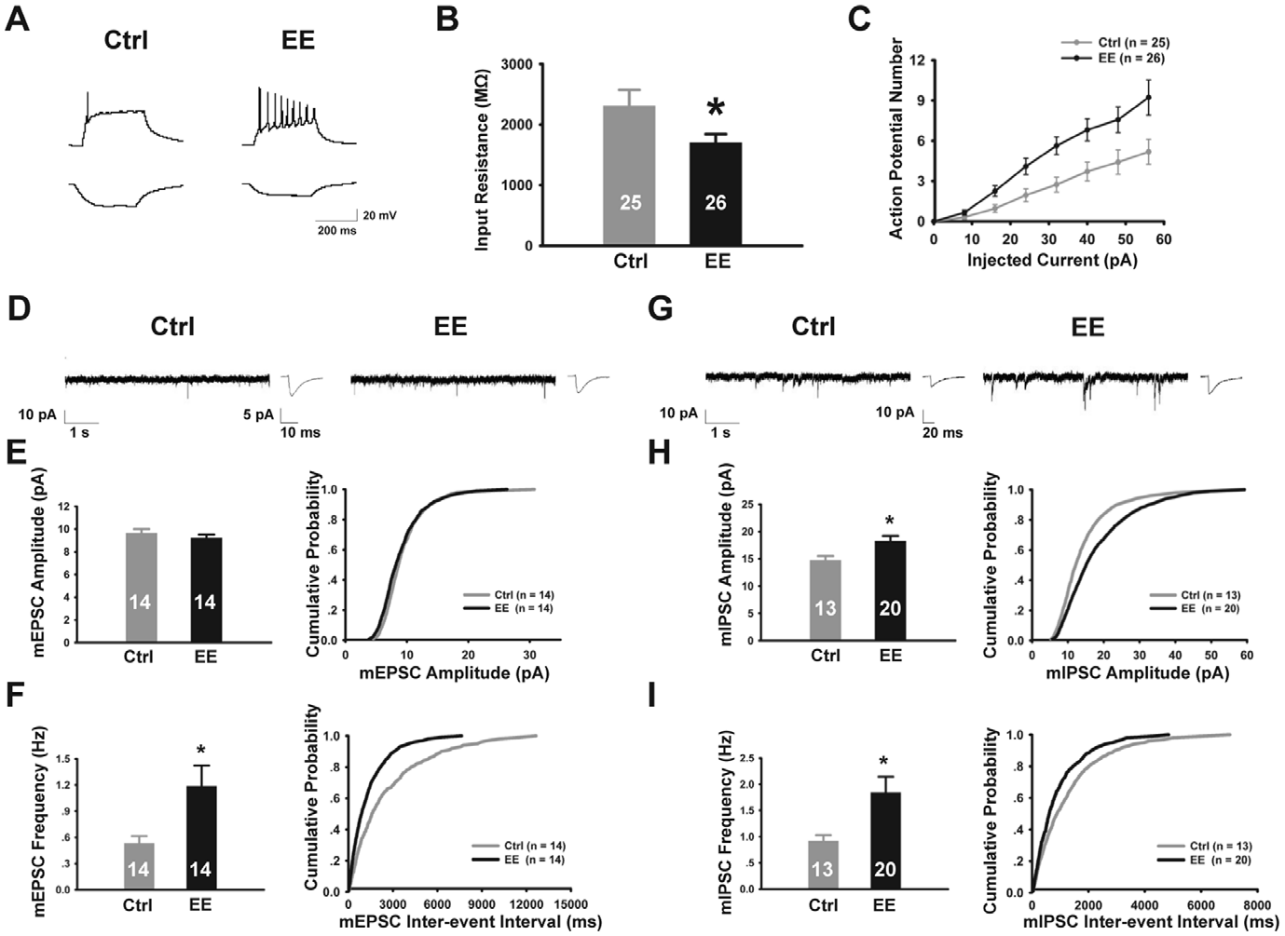
EE-rearing for 2 weeks promoted functional and synaptic maturation of dentate granule cells. (A) Top, response of dentate granule cells from control (left) and EE-reared (right) animals to depolarizing current injections (56 pA, 300 ms). Bottom, granule cell responses to hyperpolarizing current injections (−16 pA, 300 ms). (B) The average input resistance of granule cells from EE-reared mice (1703.3±139.9 MΩ) was significantly lower than that of control mice (2308.4±263.5 MΩ, N = 3 mice each, *P*<0.05). (C) Square wave depolarizing current steps (−16 to 56 pA, 300 ms) were given in 8 pA increments. Quantitation showed that more action potentials were elicited by equivalent current steps in EE-reared mice as compared to controls. (D) Sample mEPSC recordings and average mEPSC waveforms from control and EE-reared mice. (E) EE-rearing did not significantly affect average mEPSC amplitude. (F) EE-rearing significantly increased average mEPSC frequency (Ctrl: 0.53±0.08 Hz; EE: 1.19±0.23 Hz, N = 3 mice each, *P*<0.05), and resulted in a left shift of the cumulative probability distribution (*P*<0.001). (G) Sample mIPSC recordings and average mIPSC waveforms from control and EE-reared mice. (H) The average mIPSC amplitude of EE-reared mice was significantly higher (Ctrl: 14.72±0.78 pA, EE: 18.26±0.94 pA, *P*<0.05), also shown as a right shift of the cumulative probability distribution (*P*<0.01). (I) The average mIPSC frequency of EE-reared mice was significantly increased (Ctrl: 0.92±0.11 Hz, N = 3 mice; EE: 1.84±0.30 Hz, N = 4 mice, *P*<0.01), also shown as a left shift of the cumulative probability distribution (*P*<0.05). Error bars represent s.e.m., and “*n*”, as indicated inside bar graphs, represents the number of cells recorded; 3–4 mice from age-matched litters were used for each experiment; *P<0.05.

To more directly examine the effects of EE-rearing on the functional properties of neurons in the dentate gyrus, we performed whole cell patch clamp recordings from granule cells and found that EE-rearing significantly reduced the average input resistance of granule cells (Figure 5A,B). Since input resistance is a measure of the cell membrane area and ion channel density, its reduction is generally considered a hallmark of neuronal maturation [19], [40], [41], [42]. Consistent with a lowered input resistance of granule cells from EE-reared mice, greater number of action potentials were elicited following step depolarizing current injections (Figure 5A,C), indicating functionally more mature neurons.

We next examined the synaptic properties of granule cells from control and EE-reared mice at P14. We found that both glutamatergic and GABAergic synaptic transmission were significantly enhanced in P14 EE-reared mice, as compared to age-matched controls (Figure 5D–I). Specifically, in terms of glutamatergic synapses, we found a significant increase in the frequency of miniature excitatory postsynaptic currents (mEPSCs), with no significant changes in mEPSC amplitude (Figure 5D–F), suggesting an increase in the number of excitatory synapses and/or an increase in their transmitter release efficacy, without changes in the number of post-synaptic receptors per individual synapse. In terms of GABAergic synapses, we found significant enhancement in both the amplitude and frequency of miniature inhibitory postsynaptic currents (mIPSCs) (Figure 5G–I), suggesting increases both in the number of inhibitory synapses and/or their efficacy, as well as increased number of receptors per synapse. Supporting an increase in total receptor number, we observed elevated level of both GluR2 and GABA_A_Rγ2 in EE-reared mice by Western blotting (Figure 4F–H). Increase in the number of excitatory synapses per neuron is also supported by the observed increase in spine density (Figure 4A–E). Together, these results demonstrate that environmental enrichment accelerates the development of both excitatory and inhibitory synaptic inputs onto dentate granule cells.

## Discussion

Using neonatal EE-rearing as a paradigm, we investigated the effects of natural sensory stimulation on development and maturation of granule cells in the dentate gyrus. We found accelerated morphological maturation of granule cells during the first two postnatal weeks, as measured by the expression of a number of marker proteins. Furthermore, using a combination of whole cell patch-clamp recordings, Western blotting and Golgi staining, we found enhanced glutamatergic and GABAergic synaptic transmission, as well as increased receptor expression and synapse number. Together, these results provide evidence for the important role of natural environmental stimulation on the morphological, functional and synaptic maturation of dentate granule cells during the early postnatal period.

Our conclusions regarding the accelerated maturation of neurons in the dentate gyrus following environmental enrichment are based a combination of parameters assaying different aspects of neuronal development. The most complete data set is from the P14 time point, where we observed elevated expression of neuronal markers including NeuN, calbindin and MAP2 (Figure 3), together with increased spine density (Figure 4A–E). In terms of synaptic function, we found elevated number of both excitatory and inhibitory synapses, as shown by increased frequency of miniature post-synaptic currents (Figure 5D–I), and supported by elevated receptor expression (Figure 4G–H). The morphological parameters, including the expression level of NeuN, Calbindin and MAP2, were also assayed at P21 (Figure 3). In the case of Calbindin and MAP2, the results followed the same trend as at P14, albeit with a smaller magnitude of increase. For NeuN, where the increase at P14 was smaller in magnitude to start with, there were no detectable differences between the control and EE groups at P21. The plateauing of the EE effect or the “catching up” by the control group during developmental is similar to our results obtained in other regions of the hippocampus [34] and supports our conclusion regarding accelerated development and maturation through EE-rearing. The DCX results (Figure 2) are slightly more complicated, as the expression of DCX, a marker for young neurons, first increases and then decreases during neuronal maturation [33]. At P4, when all neurons are young, EE-rearing significantly elevated DCX expression (Figure 2A–C, E). By P7 and more significantly at P14, a reduction in DCX expression was observed (Figure 2A–B, D, F–G), likely due to an accelerated rate of neuronal maturation. By P21, most neurons in the dentate gyrus, in both control and EE mice, probably have reached their mature state of DCX expression, with newborn neurons being a sufficiently small percentage of the total to not affect the global level of DCX expression (Figure 3A–B, H).

The difference between the high rate of new neuron formation in the dentate gyrus during early postnatal development, especially in the first postnatal week [7], [9], and its much lower rate later in adulthood makes different methods of quantification more appropriate for each developmental time window. For studying adult neurogenesis, the standard in the field is to count the number BrdU-labeled newborn neurons using stereological methods [43]. With its advantage of unbiased sampling, stereology is ideal for measuring absolute cell numbers within a defined brain region, such as the dentate gyrus. An alternative approach is to measure relative protein intensity using quantitative immunohistochemistry [44]. The former has the advantage of an absolute cell count, while the latter measures semi-quantitatively relative changes in protein expression level over an entire brain region. For studies of early dentate gyrus development, such as ours, the latter quantification method is likely more appropriate, as cells in the entire dentate gyrus are at a similar stage of development and can be examined as a group. Especially for cytoskeletal-associated markers, such as DCX and MAP2, which can be highly overlapping and intertwined between neighboring cells, having a total measurement for the entire brain slice is perhaps more representative overall. In our assays, we took care to normalize the number of cells across different brain slices using the area of the nuclei, and compared brain slices from the EE-reared group with those of the paired control group, co-processed through all steps of perfusion, immunohistochemistry, confocal microscopy and image analysis, to obtain relative quantitation of protein expression level. Results obtained from these measurements enabled us to quantify changes in the average maturation level of granule cells in control and EE-reared mice, in line with the results of our electrophysiological and biochemical assays.

In the context of existing knowledge of the effects of environmental stimulation on granule cell development and maturation in the dentate gyrus, what new information does our current work provide? First, we demonstrated that EE-rearing during the first two weeks of postnatal development was sufficient to increase excitatory inputs onto dentate granule cells (Figure 5D–F). Previous work had shown that voluntary running increased the magnitude of long-term potentiation in the dentate gyrus [17]. Our results demonstrating an increase in the frequency of mEPSCs (Figure 5D–F), increased level of PSD95 and GluR2 (Figure 4F, G) and higher spine density (Figure 4A–E) in EE-reared mice provided a potential mechanism through which this environmentally-induced increase in synaptic plasticity may occur. Consistent with previous reports of the effect of EE-rearing on the morphological maturation of newborn neurons in the adult dentate gyrus [45], [46] and with our electrophysiological findings, we observed increased expression of NeuN, Calbindin and MAP2 at P14 (Figure 3) and accelerated peaking of DCX expression during the first postnatal week (Figure 2). The effects of environmental enrichment on promoting neuronal development and maturation are not limited to the dentate gyrus, as similar effects were observed in CA1 region of the hippocampus (Figure S1) [34].

We also showed that EE-rearing significantly increased inhibitory synaptic inputs onto granule cells, as measured by increased frequency and amplitude of mIPSCs (Figure 5G–I) and elevated level of GABA_A_Rγ2 (Figure 4H). Coordinated and balanced development of excitatory and inhibitory synaptic transmission is critical for building a stable neuronal network [47]. Whether similarly balanced development of glutamatergic and GABAergic synaptic transmission occur in newborn neurons of the adult would be interesting, as in the adult dentate gyrus there would already be plenty of inhibition in the existing network to which newborn neurons integrate. The importance of inhibition in the dentate gyrus for proper brain function is underscored by the occurrence of spontaneous seizures or lowered seizure threshold in mouse mutants with defects in dentate gyrus development [12], [13], [14].

Another feature of interest from our results is the rapid time course through which environmental stimulation accelerated neuronal development of granule cells. Previous work had already shown that, in comparison to development in the early postnatal dentate gyrus, adult-born granule cells exhibited a more prolonged time course of neuronal maturation [20], [21], [22]. In fact, adult-derived neurons did not acquire activity-dependence until after two weeks of development [48]. In contrast, neurons from the developing dentate gyrus not only responded to activity during the first two postnatal weeks (Figure 1), they also displayed significantly accelerated morphological, functional and synaptic maturation within the first two weeks of exposure to an enriched environment. Since the vast majority of granule cells in the dentate gyrus are born during the early postnatal period, our results suggest that their *en masse* development is likely to be mutually stimulating, thereby enabling faster development and maturation of the entire group in response to environmental stimulation.

## Materials and Methods

### Animals and EE-rearing paradigm

All animal studies were approved by the Institutional Animal Care and Use Committee of the Institute of Neuroscience, Chinese Academy of Sciences (Shanghai, China). The approval number is: NA-100412.

The EE-rearing protocol was essentially as previously described [34]. Briefly, C57BL/6 timed pregnant mice were randomly assigned to the standard or EE group 4–7 days prior to delivery. For the spine density measurements in Figure S1, GFP-M transgenic mice, expressing GFP under the Thy-1 promoter, were used [49]. All mice were exposed to a 12 h light/12 h dark cycle with food and water provided *ad libitum* from the cage lid. One dam and its litter were placed in standard control cages (32.5×21×18.5 cm), while two dams with a combined litter size of 10 pups or more were placed in EE cages (50×36×28 cm). Each EE cage also contained objects of various shapes and textures (wooden or plastic houses, igloos, tunnels, and wood blocks) repositioned daily and completely substituted weekly, as well as a spice cube containing different spices and additional bedding materials (shredded paper, cotton, textiles of various textures) to provide olfactory and somatosensory stimulations. Excluding space occupied by the toys, the average amount of floor space available per mouse is roughly equivalent between cage types. Both male and female pups were used in roughly equal proportions and no significant differences were observed between them (Tables S1, S2, S3, and S4).

### Immunohistochemistry, morphology analyses and data quantification

Age-matched control and EE-reared mice were deeply anaesthetized with 0.7% sodium pentobarbital and perfused with 0.9% saline, followed by 4% paraformaldehyde (PFA) in phosphate-buffered saline. Brain samples were postfixed with 4% PFA and equilibrated in 30% sucrose. Coronal sections of 30 µm were cut with a Leica CM1900 cryostat (Wetzlar, Germany). Sections were incubated in blocking solution containing 5% bovine serum albumin (BSA) and 0.1% TritonX-100, and subsequently with primary antibodies overnight in 3% BSA at 4°C. The following antibodies were used: rabbit anti-calbindin (1∶500; Sigma), rabbit anti-DCX (1∶300; Cell Signaling), mouse anti-NeuN (1∶100; Millipore), and rabbit anti-MAP2 (1∶300; Millipore). Corresponding Alexa Fluor-conjugated (488 or 568 nm) secondary antibodies were used at 1∶1000 (Invitrogen). Nuclei were labeled with TO-PRO-3 (1∶5000; Invitrogen). Golgi staining was carried out according to the manufacturer's instructions (FD NeuroTechnologies).

Images (Z stacks at 1 µm intervals) were acquired on a Zeiss Pascal laser scanning microscope (Jena, Germany) with a 40× oil immersion Neofluor objective (N.A. = 1.3). For analysis of immunostaining intensity, the granule cell-containing region of the dentate gyrus was outlined from projected Z-stacks in the TO-PRO-3 channel in ImageProPlus software (MediaCybernetics). The staining intensity of each neuronal marker was calculated by normalizing its total intensity (mean intensity×total area) to the total area of TO-PRO-3. The values for EE-reared mice were then normalized to those of its age-matched and paired control. Each pair of control and EE mice were co-processed for all steps of perfusion, immunohistochemistry, confocal microscopy and image analyses. Anatomically matched sections were imaged from each pair of animals. For analysis of spine density, Golgi-impregnated dendritic segments from both the superpyramidal and infrapyramidal blades were used, and images were analyzed blindly. For spine density analyses in CA1 region of the hippocampus, GFP fluorescence of GFP-M mice was used to label the morphology of CA1 pyramidal neurons. Dendrite length was measured in ImageProPlus, and spine numbers were manually counted. All results were statistically tested using two-tailed Students t-test with equal variance and shown as mean ± s.e.m.; “*n*” represents the number of images analyzed, while “N” represents the number of mice. At least 3 pairs of mice from different litters were analyzed per neuronal marker.

### Electrophysiology

Acute hippocampal slices were prepared as previously described [34]. Whole-cell voltage-clamp recordings were made from granule cells of the dentate gyrus (no specific region favored) using a MultiClamp 700B amplifier (Molecular Devices, Sunnyvale, CA, USA). Signals were filtered at 2 kHz and sampled at 10 kHz. For miniature excitatory postsynaptic current (mEPSC) recordings, the internal solution contained (in mM) CsMeSO_4_ 100, CsCl 25.5, HEPES 10, NaCl 8, EGTA 0.25, glucose 10, MgATP 4, Na_3_GTP 0.3 (pH 7.3, 280–290 mOsm), and neurons were held at −70 mV; 50 µM picrotoxin and 0.5 µM tetrodotoxin (TTX) were added to ACSF to block GABA_A_ and Na^+^ currents respectively. For mIPSC recordings, a high chloride internal solution containing (in mM) CsCl 110, NaCl 10, MgCl_2_ 5, EGTA 0.6, MgATP 2, Na_3_GTP 0.2, HEPES 40 (pH 7.25, 290–295 mOsm) was used, and cells were held at −60 mV; 10 µM NBQX and 0.5 µM TTX were added to ACSF to block AMPA and Na^+^ currents respectively.

Depolarizing or hyperpolarizing current steps (300 ms) were used to elicit action potentials and measure input resistance, respectively. The steady state current was used for input resistance measurement. Action potential number versus injected current curves was plotted by measuring the average number of action potentials during depolarizing current injections of increasing magnitude. The internal solution for this experiment contained (in mM): K-gluconate 110, KCl 20, HEPES 20, MgCl_2_ 5, EGTA 0.6, MgATP 2, Na_3_GTP 0.2 (pH 7.3, 280–290 mOsm). All salts and drugs were obtained from Sigma or Tocris, except for TTX, which was obtained from the Fisheries Science and Technology Development Company of Hebei Province, China.

Series and input resistances were continually monitored, and data were not included if the series resistance changed by more than 20%. mPSCs were analyzed using MiniAnalysis software (Synaptosoft), other data using Clampfit. The amplitude threshold for mEPSCs and mIPSCs were respectively 5 pA and 6 pA. Results were statistically tested using two-tailed Students t-test with equal variance; cumulative distributions were tested against each other using the Kolmogorov-Smirnov two sample test (Synaptosoft). For cumulative distribution plots, 100 events per cell were used. Results are shown as mean ± s.e.m, “*n*” represents the number of neurons, and “N” represents the number of mice. At least 3 animals from different litters were analyzed per experimental condition. The P14 group included P13–15 mice.

### Western blots

The entire dentate gyrus was dissected, and Western blots were carried out as previously described [34]. Membrane fractions were used, except for BDNF (whole sample) and c-fos (nuclei). The following antibodies were used: rabbit anti-GABA_A_Rγ2 (1∶2000; Alomone), mouse anti-GluR2 (1∶1000; Millipore), rabbit c-fos (1∶20,000; Santa Cruz), rabbit anti-BDNF (1∶500; Santa Cruz), mouse anti-PSD95 (1∶1000; Sigma), mouse anti-GAPDH (1∶10000; Kangchen) and mouse anti-TBP (1∶500; Abcam). Results were quantified using ImageJ software (NIH Image) and statistically analyzed using the Mann-Whitney rank sum test (SigmaPlot). Results are shown as mean ± s.e.m, and “*n*” represents the number of age-matched litter pairs.

## Supporting Information

Figure S1 EE-rearing upregulated MAP2 staining and increased spine density in CA1 region of the hippocampus at P14. (A) Example images of MAP2 staining in pyramidal neurons of the hippocampal CA1 region in Ctrl and EE-reared mice. (B) EE-rearing significantly increased MAP2 immunoreactivity in the pyramidal layer of the CA1 region (1.41±0.09, N = 6 mice each, *P*<0.001). (C) Example images of dendritic spines of CA1 hippocampal pyramidal neurons, scale bar is 5 µm. (D) Quantitative analysis of spine density showed significantly higher dendritic spines in EE-reared mice (Ctrl: 8.74±0.34, N = 4 mice; EE:10.30±0.34, N = 5 mice, *P*<0.01).
(TIF)

Table S1 EE significantly reduced DCX level in both male and female mice at P14.
(DOC)

Table S2 No significant differences in DCX expression level between male and female mice at P14 in either Ctrl or EE conditions.
(DOC)

Table S3 EE significantly increased calbindin level in both male and female mice at P14.
(DOC)

Table S4 No significant differences in calbindin expression level between male and female mice at P14 in either Ctrl or EE conditions.
(DOC)
