# Supplementary figures and images for: Early Natural Stimulation through Environmental Enrichment Accelerates Neuronal Development in the Mouse Dentate Gyrus
Source: PLoS One. 2012 Jan 25;7(1):e30803. doi: 10.1371/journal.pone.0030803 (PMC3266290; doi:10.1371/journal.pone.0030803)

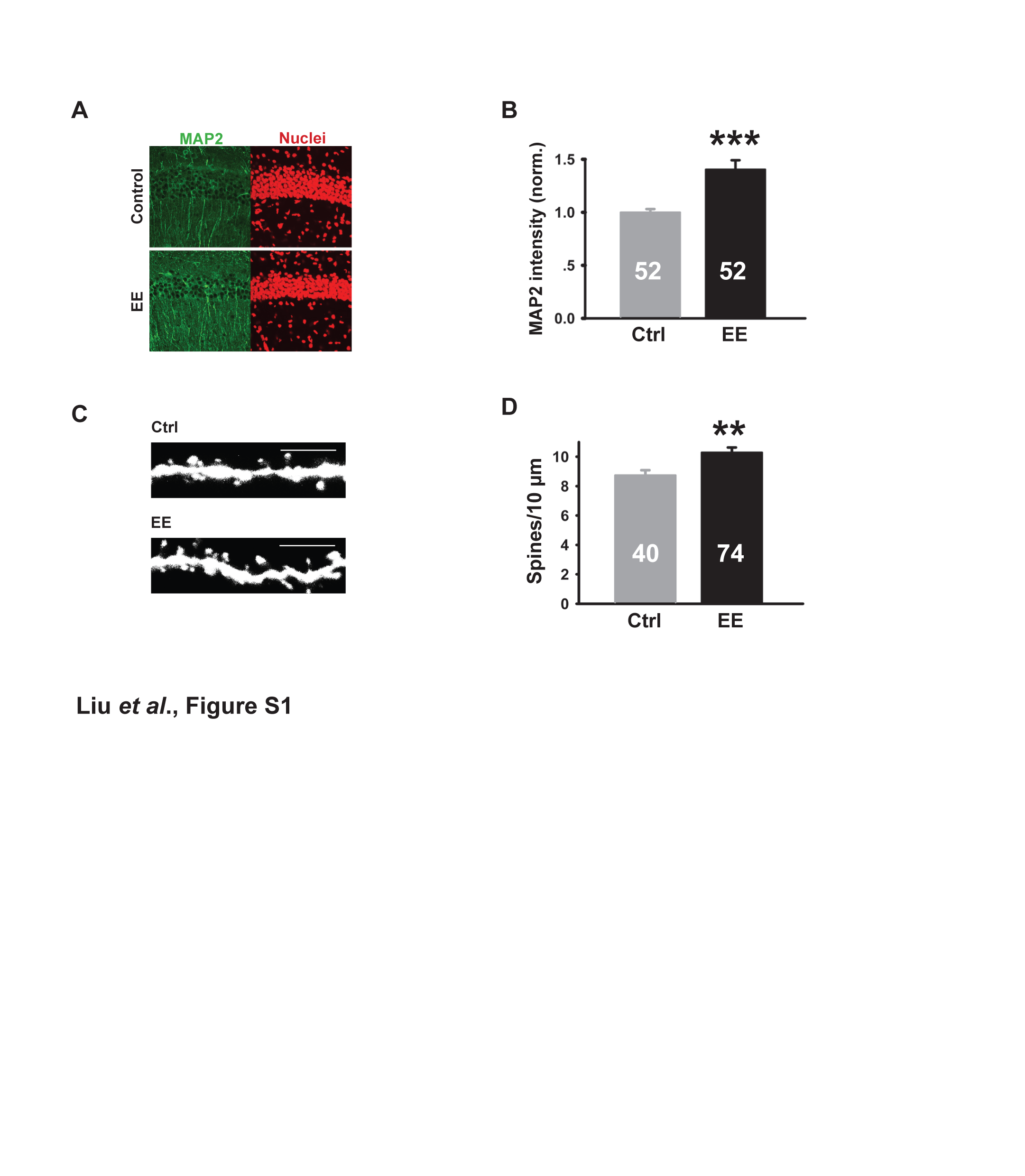

Supplement: Figure S1 — EE-rearing upregulated MAP2 staining and increased spine density in CA1 region of the hippocampus at P14. (A) Example images of MAP2 staining in pyramidal neurons of the hippocampal CA1 region in Ctrl and EE-reared mice. (B) EE-rearing significantly increased MAP2 immunoreactivity in the pyramidal layer of the CA1 region (1.41±0.09, N = 6 mice each, P<0.001). (C) Example images of dendritic spines of CA1 hippocampal pyramidal neurons, scale bar is 5 µm. (D) Quantitative analysis of spine density showed significantly higher dendritic spines in EE-reared mice (Ctrl: 8.74±0.34, N = 4 mice; EE:10.30±0.34, N = 5 mice, P<0.01). (TIF) [file pone.0030803.s001.tif]
