# Supplementary material for: Early Natural Stimulation through Environmental Enrichment Accelerates Neuronal Development in the Mouse Dentate Gyrus
Source: PLoS One. 2012 Jan 25;7(1):e30803. doi: 10.1371/journal.pone.0030803 (PMC3266290; doi:10.1371/journal.pone.0030803)
Supplement: Table S2 — No significant differences in DCX expression level between male and female mice at P14 in either Ctrl or EE conditions. (DOC) [file pone.0030803.s003.doc]

**Liu *et al.,* Supplementary Tables**

**Table S2: No significant differences in DCX expression level between male and female mice at P14 in either Ctrl or EE conditions.**

|  | **Ctrl** | **EE** |
| --- | --- | --- |
| **Male** | 1.00±0.06 | 1.00±0.11 |
| **Female** | 1.09±0.08 | 0.85±0.04 |
| **P (t-test)** | 0.37 | 0.11 |
