# Supplementary material for: Early Natural Stimulation through Environmental Enrichment Accelerates Neuronal Development in the Mouse Dentate Gyrus
Source: PLoS One. 2012 Jan 25;7(1):e30803. doi: 10.1371/journal.pone.0030803 (PMC3266290; doi:10.1371/journal.pone.0030803)
Supplement: Table S3 — EE significantly increased calbindin level in both male and female mice at P14. (DOC) [file pone.0030803.s004.doc]

**Liu *et al.,* Supplementary Tables**

**Table S3: EE significantly increased calbindin level in both male and female mice at P14.**

|  | **Ctrl** | **EE** | **P (t-test)** |
| --- | --- | --- | --- |
| **Male** | 1.00±0.06 | 1.78±0.11 | <0.001 |
| **Female** | 1.00±0.05 | 1.95±0.13 | <0.001 |
| **Total** | 1.00±0.03 | 1.84±0.12 | <0.001 |
