# Supplementary material for: Early Natural Stimulation through Environmental Enrichment Accelerates Neuronal Development in the Mouse Dentate Gyrus
Source: PLoS One. 2012 Jan 25;7(1):e30803. doi: 10.1371/journal.pone.0030803 (PMC3266290; doi:10.1371/journal.pone.0030803)
Supplement: Table S4 — No significant differences in calbindin expression level between male and female mice at P14 in either Ctrl or EE conditions. (DOC) [file pone.0030803.s005.doc]

**Liu *et al.,* Supplementary Tables**

**Table S4: No significant differences in calbindin expression level between male and female mice at P14 in either Ctrl or EE conditions.**

|  | **Ctrl** | **EE** |
| --- | --- | --- |
| **Male** | 1.00±0.06 | 1.00±0.07 |
| **Female** | 0.88±0.09 | 0.97±0.12 |
| **P (t-test)** | 0.28 | 0.78 |
